# Charting the future: A multi-setting medical chart study of advance care planning in Parkinson's disease and atypical Parkinsonism

**DOI:** 10.1177/1877718X261495736

**Published:** 2026-10-13

**Authors:** Herma Lennaerts-Kats, Bauke W. Dijkstra, Anke Elbers, Bastiaan R. Bloem, Marieke C. Groot, Jenny T. van der Steen, Marjan J. Meinders

**Affiliations:** 1 Department of Primary and Community Care and Radboudumc Alzheimer Center, 6034 Radboud university medical center, Geert Grooteplein Noord 21, P.O. Box 9101 Nijmegen, The Netherlands; 2 Department of Donders Institute for Brain, Cognition and Behaviour; Department of Neurology, Center of Expertise for Parkinson & Movement Disorders; Radboudumc, Nijmegen, the Netherlands; 3 Research Centre Innovations in Care, 6985 Rotterdam University of Applied sciences, Rotterdam, The Netherlands; 4 Department of Public Health and Primary Care, 4501 Leiden University Medical Center, Hippocratespad 21, Building 3, P.O. Box 9600 Leiden, The Netherlands; 5 Cicely Saunders Institute, King's College London, London, UK

**Keywords:** advance care planning, Parkinson's disease, atypical Parkinsonism, palliative care, end-of-life care, medical record

## Abstract

**Background:**

Advance Care Planning (ACP) enables people with progressive diseases to document and communicate future treatment preferences. For Parkinson's disease (PD) and atypical parkinsonism (AP), ACP is particularly important because of disease complexity, symptom burden, the progressive nature of the disease, and cognitive decline, yet its implementation across care settings remains unclear.

**Methods:**

We conducted a medical chart review of 189 deceased patients with PD or AP who died in nursing homes (n = 91), hospitals (n = 58), or at home (n = 40) in the Netherlands. For patients who died at home, data were obtained from their general practice records. Data on treatment preferences, care discussions and palliative care goals were extracted at one year and six weeks before death.

**Results:**

Documentation of treatment preferences increased towards death but differed markedly between settings. Nursing homes recorded resuscitation and ICU limitations early and comprehensively, whereas hospitals made major updates only in the final weeks of life. In general practices, ACP documentation was limited. Nursing homes and hospitals more frequently addressed disease-specific scenarios, palliative sedation and end-of-life issues, while general practices more often reported preferred place of death and euthanasia. Palliative care goals in nursing homes covered all domains, while in hospitals and general practices they mainly related to physical aspects.

**Conclusion:**

ACP remains inconsistently documented and frequently delayed until the final weeks of life, when crises or cognitive decline may hinder meaningful participation. Earlier, iterative ACP—supported by clinician training, PD-specific guidance, and system-level measures such as ACP prompts in electronic medical records—could foster a more proactive and person-centered palliative approach aligned with patient values throughout the disease trajectory.

## Plain language summary title

Planning future care for people with Parkinson's disease and Atypical parkinsonism: what medical records tell us

## Plain language summary

People with Parkinson's disease and related conditions often face complex health problems as their disease progresses. Thinking ahead and talking about future care wishes—also called Advance Care Planning—can help ensure that care at the end-of-life matches what people want. However, it is not well known how often and how well these plans are recorded in everyday healthcare.

In this study, we reviewed medical records of 189 people with Parkinson's disease or atypical parkinsonism who had died in the Netherlands. We looked at records from nursing homes, hospitals, and general practitioner (GP) practices. We examined what was written about care wishes, such as preferences about resuscitation, hospital admission, place of death, and comfort-focused care. We compared what was documented one year before death and six weeks before death.

We found that information about future care wishes was more often recorded as people approached the end-of-life, but this differed strongly between care settings. Nursing homes usually documented care preferences early and in more detail. Hospitals often recorded or updated care wishes only in the final weeks of life, usually during acute illness. GP records contained relatively little information about advance care planning. In addition, most records focused on physical care, while emotional, social, and existential needs were less often documented.

Our findings suggest that planning future care for people with Parkinson's disease often happens late and inconsistently. Starting these conversations earlier and revisiting them over time may help ensure that care better reflects patients' wishes throughout the course of the disease.

## Introduction

Advance Care Planning (ACP) is a structured communication process in which healthcare professionals engage with patients to discuss and document future healthcare preferences, particularly in the context of progressive and life-limiting conditions.^1^ For patients with Parkinson's disease (PD) and atypical parkinsonism (AP), ACP is of particular importance due to the disease's inevitable progression and complex phenotype, including motor and non-motor symptoms that can significantly impact quality of life and decision-making capacity.^2–4^

ACP has been shown to improve the quality of end-of-life care by aligning treatment with patient preferences, reducing non-beneficial hospitalizations, and enhancing communication among patients, families, and healthcare providers.^5–8^ A recent meta-review reinforces these benefits, reporting increased documentation of care preferences, more frequent care in the preferred setting, and moderate-certainty evidence for greater concordance between stated wishes and delivered care.^9^ Effects on healthcare utilization and patient-reported outcomes remain mixed, likely due to heterogeneity across studies. However, despite its proven benefits, ACP remains inconsistently integrated into clinical practice, with variations in timing, documentation, and follow-up limiting its overall impact.^10–12^

In the Netherlands, general practitioners play a pivotal role in coordinating care for patients with chronic illnesses, yet studies suggest that ACP documentation in primary care remains inconsistent. This was confirmed in a recent nationwide study, which analyzed over 199,000 medical records from Dutch general practices.^13^ The study demonstrated that ACP initiation is least frequent in patients with organ failure and dementia, while patients with cancer had the highest rates of ACP documentation. Importantly, patients with PD showed ACP initiation rates comparable to other chronic disease groups, but still markedly lower than cancer, highlighting systemic disparities in ACP practice across diagnoses. These findings align with a study that found that although most people with PD expressed a willingness to plan ahead, awareness and formal engagement in ACP was low. In a UK-based mixed methods study, 76% of respondents had never heard of ACP, yet nearly 70% indicated interest in learning more. Most participants had arranged legal or practical matters such as a will or power of attorney but rarely discussed treatment preferences or end-of-life care with healthcare professionals.^12^ For people with PD or AP, timely ACP may be of particular importance, because many develop progressive communication and cognitive problems in the late stage. Important treatment decisions are difficult to make when an affected person has lost the capacity to communicate. Family caregivers and healthcare professionals are often left trying to determine a person's wishes for care, as these wishes were not discussed or formally recorded.^4^

These findings underscore the urgency of strengthening ACP in PD and AP, where symptom progression and cognitive decline narrow the window of opportunity for ACP. The current study aimed to investigate the state of ACP documentation in these patient groups in multiple healthcare settings. Our study sought to inform strategies for improving ACP practices and ensuring that patients with PD receive care aligned with their values and wishes.

## Material and methods

### Study design

We conducted a medical record study in nursing homes, hospitals and general practices in the Netherlands. Our objective was to capture the diversity and context of ACP information in each setting, acknowledging differences in patient populations and documentation methods.

### Study setting and data extraction

We reviewed medical records of patients diagnosed with PD or Atypical Parkinsonism (AP) who had died between October 2018 and November 2023. The planned sample size was informed by previous retrospective medical record review studies^14^ reporting on palliative care and ACP documentation, as well as by feasibility considerations related to available time and resources. We therefore aimed to include approximately 150 medical records, evenly distributed across the three healthcare settings. This target was based on the anticipated number of participating institutions and the estimated number of eligible records per institution.

Nursing homes and hospitals were approached directly for participation, building on established collaborations with neurologists, PD nurses, and elderly care physicians from a prior ParkinsonSupport project.^15^ For primary care data, we utilized fully digitalized routinely collected data from 11 general practices, participating in the Radboudumc Family Medicine Network, representing a patient population of over 66,000 individuals in the eastern region of the Netherlands. All participating sites complied with relevant ethical and data protection regulations.^16^ Patients were categorized according to their place of death and the corresponding healthcare setting from which the medical record was obtained. Patients in the nursing home group died in a nursing home, patients in the hospital group died in hospital, and patients in the general practice group died at home and their general practice records were reviewed. For each included record, we extracted ACP documentation recorded during the final year of life and ACP documentation present or updated during the final six weeks of life. The six-week period was considered a second observation point within the final year of life rather than a separate non-overlapping period. Patients were not excluded if they had moved between healthcare settings during the final year of life. When ACP information had been transferred from another setting and was present in the record of the setting where the patient died, it was included in the analysis. All available medical records of deceased patients with PD or AP who met the inclusion criteria during the study period were considered for inclusion. No sampling procedure was used. Medical records were excluded only when data were insufficient for meaningful analysis, defined as having more than 80% of the required data fields missing. Furthermore, we selected healthcare settings rather than specific medical specialties, as the study aimed to capture ACP documentation in the final phase of life. Therefore, complete hospital records were reviewed irrespective of the admitting specialty. During data collection, participating institutions identified more eligible records than anticipated within the predefined study period. These additional eligible records were included in the final dataset, resulting in a total of 189 medical records. This decision was made before any analyses were performed and was based solely on the availability of additional eligible records, not on the content of the extracted data.

### Development of data extraction form

To systematically collect detailed data on ACP in the final year of life, we developed a structured data extraction form. This instrument was informed by existing literature and previously validated tools used in studies on end-of-life care in older populations and general practice settings^14,17^ The form was iteratively refined based on the first three patient records and subsequently pilot-tested by two independent researchers (HL and AE) on five additional records. Discrepancies were identified through independent data extraction and direct comparison of the completed forms. These discrepancies mainly concerned differences in interpretation of documented ACP discussions or care preferences**.** Any discrepancies were discussed and resolved, and when consensus could not be reached, a third researcher (MJM) was consulted. This resulted in consistent data interpretation. No major interrater variation was observed thereafter.

Manual data extraction was performed on medical records from nursing homes, hospitals, and general practices. Extracted data were entered into a unified database using a standardized coding framework. The final extraction form included the following components:

1. Patient characteristics and general information: age at death, sex, comorbidities, cause and place of death, and disease duration (years from diagnosis to death);
2. ACP documentation: recorded preferences and/or wishes for end-of-life care during the final year and final six weeks of life;
3. Documented care goals per palliative care domain (physical, social, existential, psychological, other personal goals);
4. Healthcare utilization in the final six weeks: hospital admissions, hospice referrals, medication use, medical interventions (e.g., artificial nutrition, fluid administration, oxygen therapy), and end-of-life interventions (e.g., palliative sedation, euthanasia).

ACP documentation was categorized into two domains: care decisions and care discussions. For care decisions, the following items were extracted and coded as *Yes*, *No*, or *Unknown*: resuscitation policy, ICU admission, hospital admission, antibiotic treatment, artificial feeding, and fluid administration. For care discussions, we extracted whether any documentation was present (*Yes/No*) regarding the following topics: disease-specific future scenarios, preferred place of death, euthanasia, palliative sedation, general end-of-life discussions, and other treatment preferences.^14,17^

### Statistical analysis

Descriptive statistics were used to summarize all outcome measures. Changes in documented care decisions between one year and six weeks before death were assessed using McNemar's test. Each decision category (*Yes*, *No*, *Unknown*) was compared against a binary variable combining the two alternative outcomes (e.g., *Yes* vs. *Not Yes*). To account for multiple comparisons across three care settings and three decision categories, a Bonferroni correction was applied, yielding an adjusted significance threshold of *p* = 0.006 (0.05/9). Similarly, changes in care discussions between one year and six weeks before death were analyzed using McNemar's test. A Bonferroni correction for three settings resulted in an adjusted alpha of *p* = 0.017 (0.05/3). All statistical tests were two-tailed. All statistical analyses were conducted using IBM SPSS Statistics version 22.

## Results

The selection procedures resulted in 189 medical records being reviewed, of which 58 were from five hospitals, 91 from three nursing homes and 40 from 11 primary care practices (Table 1). The mean age at death was 80.2 years (SD 7.4), and 68.1% were male. PD was the predominant diagnosis, representing over 70% of cases. The average disease duration was 6.4 years, and the mean number of medications was 10.9. Palliative sedation was the most frequently used end-of-life intervention overall, in nursing homes and in general practice. In hospitals, antibiotics and fluid administration were the most frequent interventions, while euthanasia did not occur.

**Table 1.** Patient characteristics.

	Nursing home (n = 91)	Hospital (n = 58)	General practice (n = 40)
**Demographics**			
Gender, n (%) male	58 (63.7)	45 (77.6)	27 (67.5)
Age at death in years, mean (SD)	79.9 (6.7)	78.3 (7.5)	83.4 (7.9)
**Diagnosis, n (%)**			
Parkinson's disease	68 (74.7)	40 (69.0)	25 (62.5)
Parkinsonism*	6 (6.6)	2 (3.4)	5 (12.5)
Progressive supranuclear palsy	5 (5.5)	2 (3.4)	3 (7.5)
Vascular parkinsonism	5 (5.5)	3 (5.2)	5 (12.5)
Multisystem atrophy	3 (3.3)	4 (6.9)	1 (2.5)
Lewy body dementia	2 (2.2)	2 (3.4)	0
Corticobasal degeneration	1 (1.1)	0	0
Other, multiple	1 (1.1)	5 (8.6)	1 (2.5)
**Other clinical characteristics, mean (SD)**			
Disease duration in years	6.9 (14.4)	6.3 (4.5)	5.8 (4.5)
Charlson comorbidity index	4.4 (11.2)	4.5 (1.7)	6.8 (2.6)
Levodopa equivalent daily dose (LEDD) in mg/day	735.5 (538.5)	702.4 (410.2)	NA**
Number of medications	11.9 (4.6)	9.5 (4.5)	10.3 (2.7)
**Medical interventions in the six weeks before death, n (%)**			
Antibiotics	41 (45.1)	40 (69.0)	13 (32.5)
Artificial feeding	4 (4.4)	8 (13.8)	2 (5.0)
Fluid administration	12 (13.2)	40 (69.0)	4 (10.0)
Palliative sedation	63 (69.2)	34 (58.6)	16 (40.0)
Euthanasia	6 (6.6)	0 (0.0)	5 (12.5)

* Parkinsonism not otherwise specified/unclear.

**Data on PD medication were incomplete, as these medications were prescribed and recorded by neurologists in the hospital setting.

Documented palliative care goals in the last six weeks of life were most frequently present in nursing home records, covering all domains of care (Table 2). Nearly all nursing home patients had physical goals recorded, and documentation in the psychological, social, and existential dimensions was also high. In hospitals, documentation was largely limited to physical aspects, while psychosocial and existential goals were rarely recorded. General practice records showed the lowest overall rates, with physical goals documented for most patients, but with limited attention to other domains.

**Table 2.** Documented goals across palliative care domains in the final 6 weeks before death.

	Nursing home		Hospital		General practice	
Domain, n (%)	PD (n = 68)	AP (n = 23)	PD (n = 40)	AP (n = 18)	PD (n = 25)	AP (n = 15)
Physical	65 (95.6)	22 (95.7)	37 (92.5)	16 (88.9)	19 (76.0)	12 (80)
Psychological	60 (88.2)	22 (95.7)	10 (25.0)	4 (22.2)	2 (8.0)	1 (6.7)
Social	49 (72.1)	17 (73.9)	24 (60.0)	8 (44.4)	5 (20.0)	3 (20.0)
Existential	47 (69.1)	15 (65.2)	2 (5.0)	4 (22.2)	2 (8.0)	1 (6.7)

When comparing patients with PD to those with AP, patterns were largely similar across settings. In both groups, physical goals were consistently documented, whereas non-physical dimensions received less attention outside nursing homes. Across all settings, differences between PD and AP were small.

### Resuscitation policy

In nursing homes, resuscitation preferences were almost universally documented as “do not resuscitate” at both timepoints, increasing from 95% at 1 year to 100% in the final 6 weeks, with “unknown” entries declining from 4% to 0% (Figure 1: Resuscitation). In hospital records, documentation improved significantly: do-not-resuscitate (DNR) preferences rose from 53% to 96% (p < 0.001), while “unknown” entries decreased from 34% to 0% (p < 0.001). GP records showed more limited changes, with DNR preferences increased modestly (50% to 60%) and “unknown” entries remaining high (40% at 1 year, 37% at 6 weeks).

**Figure 1.**
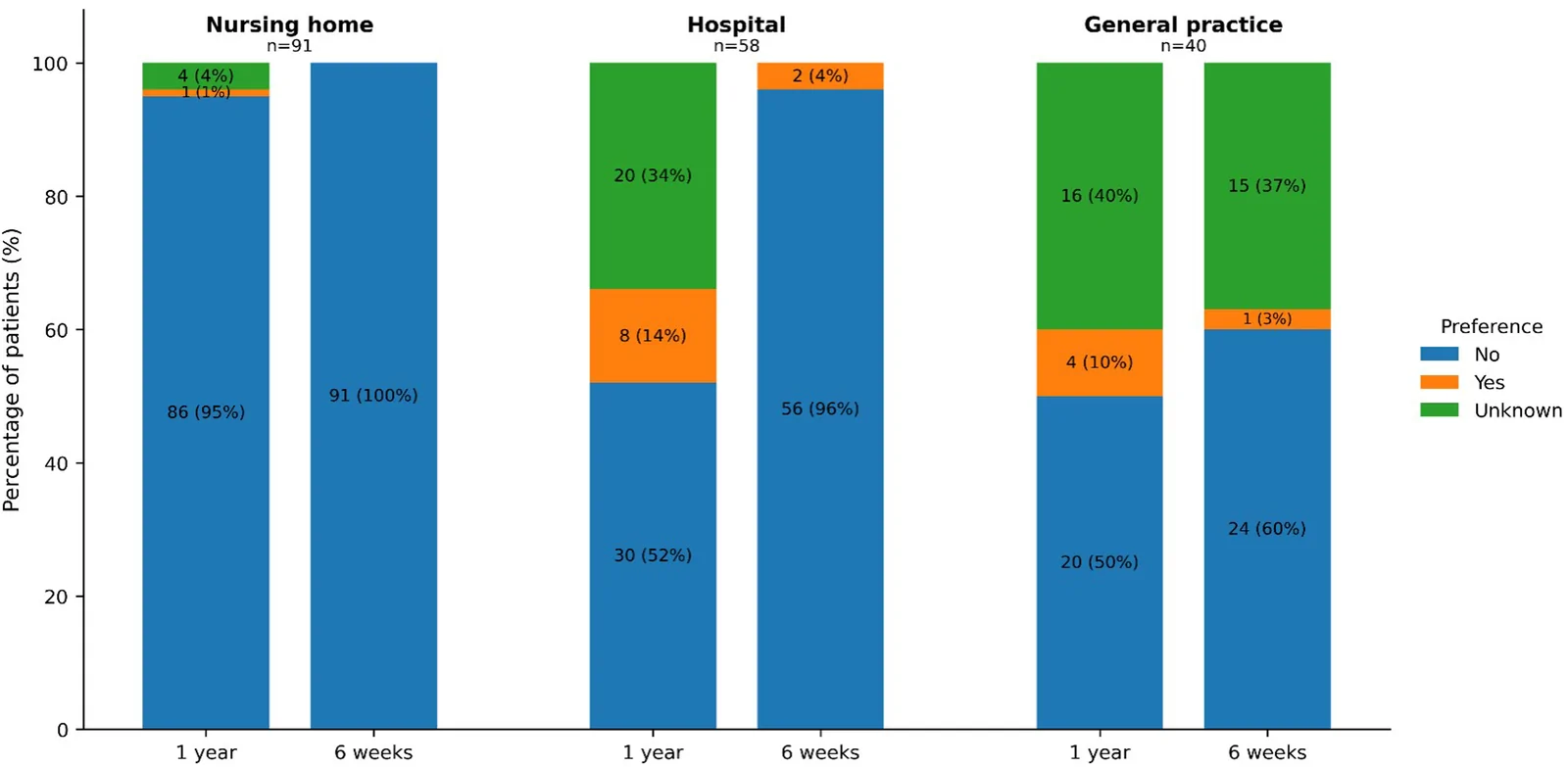
Resuscitation.

### Hospital admission

Preferences regarding a potential future hospital admission showed a marked shift in nursing homes, where documented preferences against such an admission increased from 53% at 1 year to 87% in the final 6 weeks (p < 0.001), accompanied by a decline in preferences in favor (24% to 7%, p < 0.001) and a reduction in “unknown” entries (23% to 6%) (Figure 2: Hospital admission). In contrast, hospital records remained largely unchanged, with very few preferences documented against admission (3–5%) and a stable distribution between preferences in favor (54–50%) and “unknown” (43–45%). General practice records showed limited change, with approximately half of entries remaining undocumented (55% to 45%) and only a modest, non-significant increase in preferences against admission (25% to 35%).

**Figure 2.**
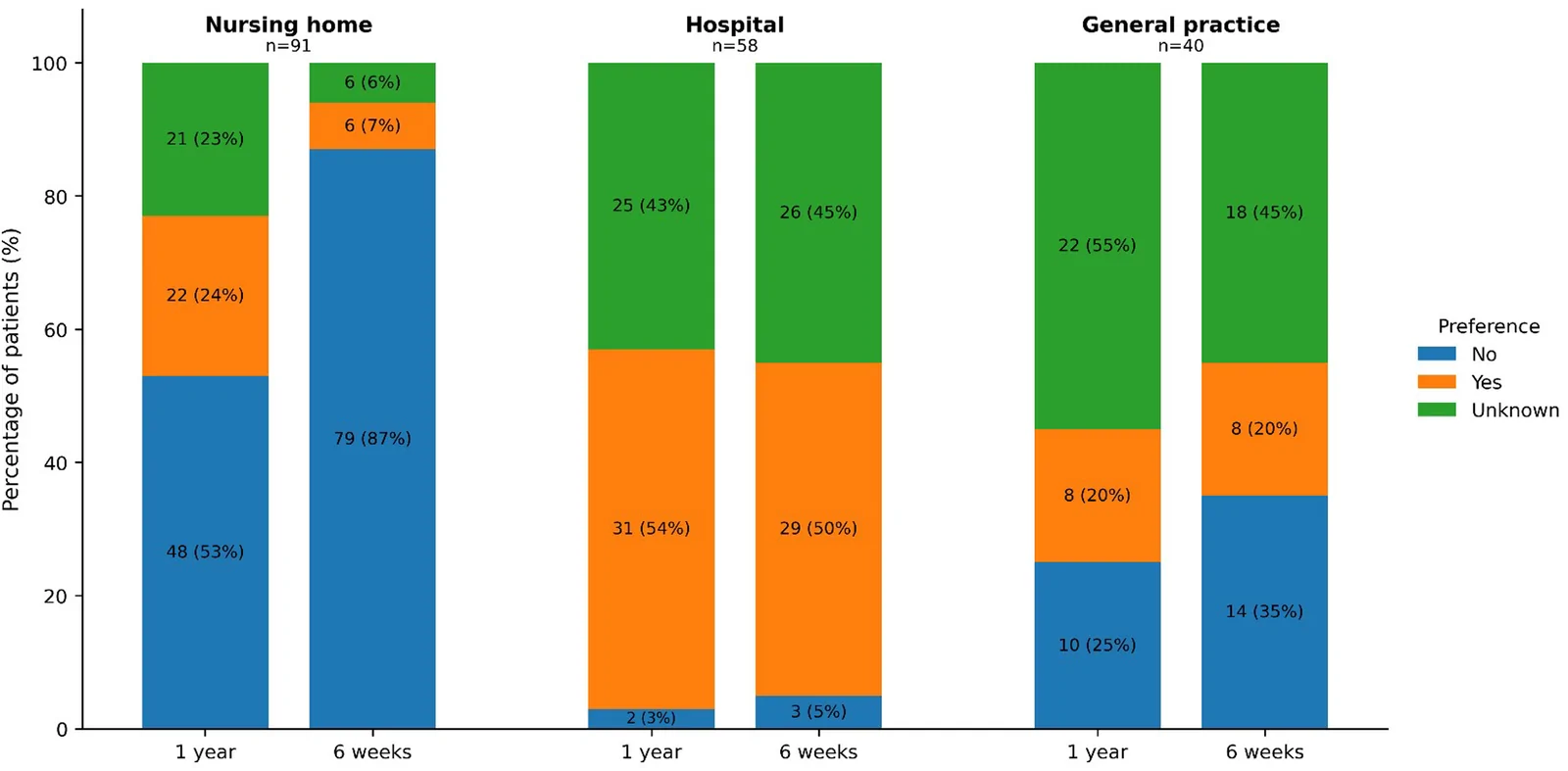
Hospital admission.

### ICU admission

Nursing home records showed a clear rise in preferences against ICU admission (73% to 91%, p < 0.001) and a concomitant decline in preferences in favor (10% to 3%, p = 0.031) (Figure 3: ICU admission). Hospital records similarly showed significant increases in documentation against ICU admission (35% to 88%, p < 0.001) and fewer “unknown” entries (46% to 5%, p < 0.001). GP records did not show significant changes, with nearly half remaining undocumented.

**Figure 3.**
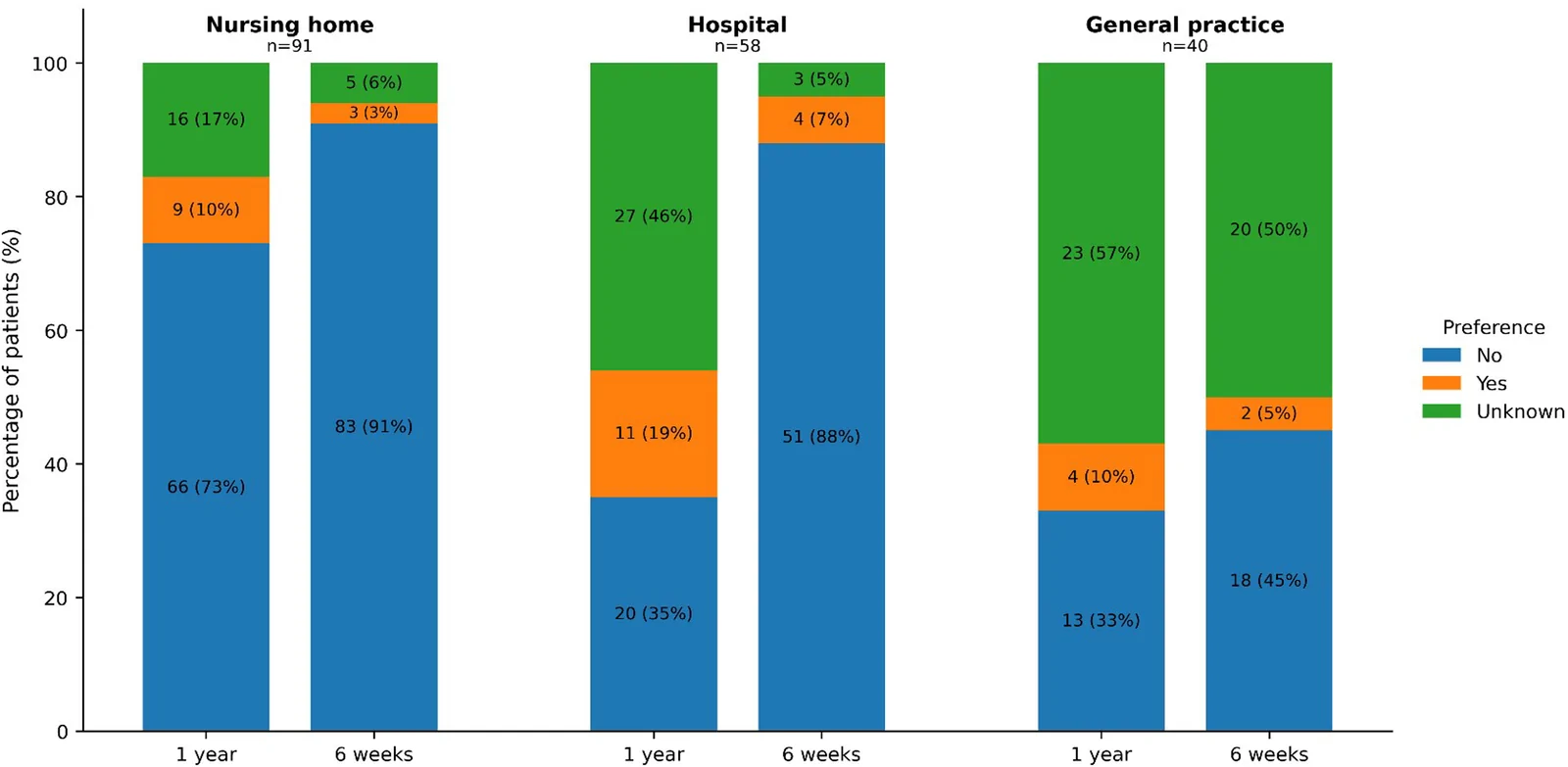
ICU admission.

### Antibiotic treatment

Nursing home records showed more frequent documentation against antibiotics (17% to 46%, p < 0.001) and fewer “unknown” entries (36% to 20%, p < 0.001) (Figure 4: Antibiotic treatment). In hospital records, preferences in favor of antibiotics increased (52% to 71%, p = 0.007), while “unknown” entries decreased (43% to 19%, p = 0.003). GP documentation remained limited, though “unknown” entries decreased (77% to 57%, p = 0.008).

**Figure 4.**
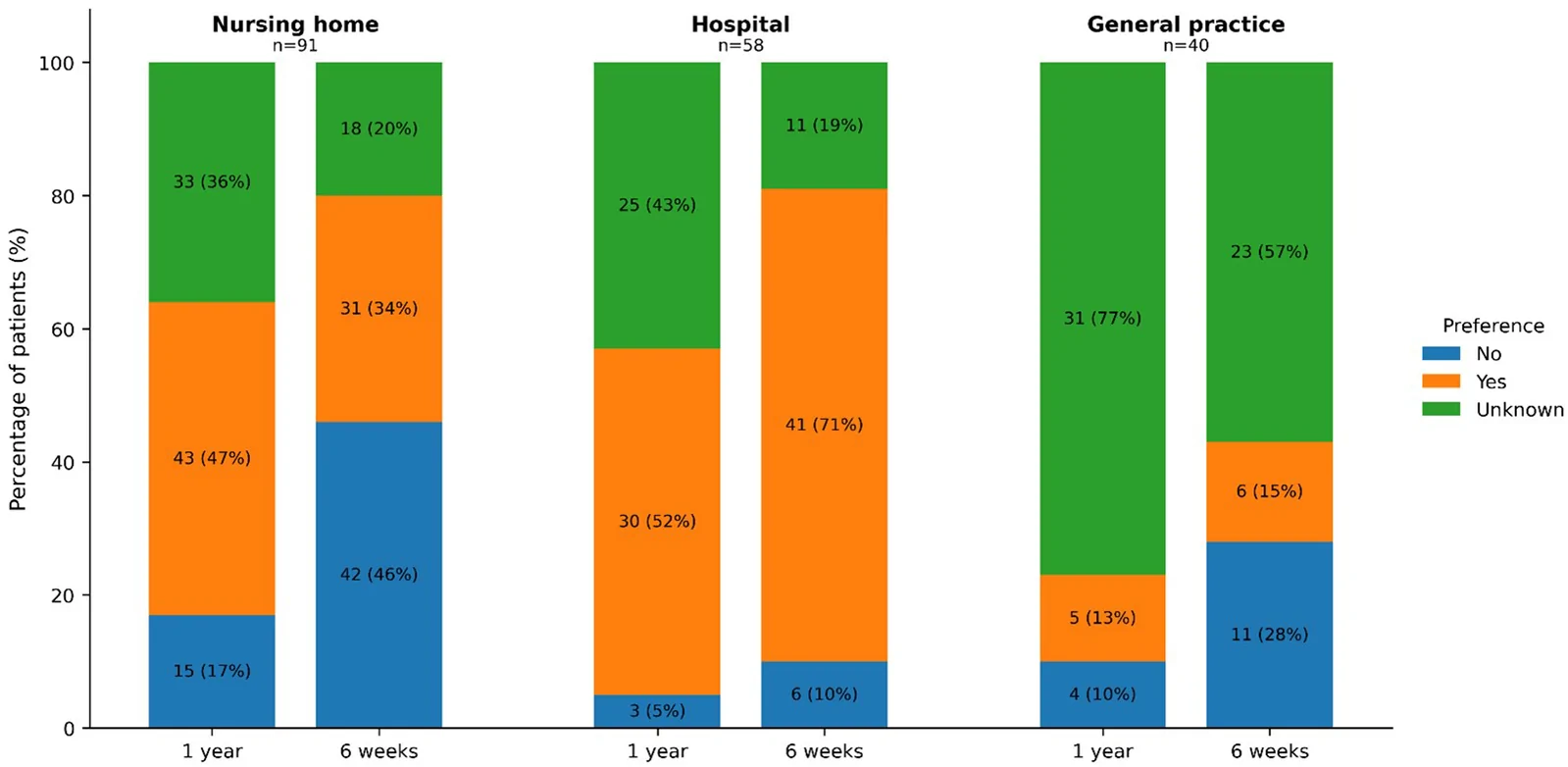
Antibiotic treatment.

### Artificial feeding and fluids

In nursing homes, documentation against artificial feeding increased substantially (40% to 58%, *p* < 0.001), with fewer “unknown” entries (42% to 32%) and a decline in positive documentation (18% to 10%) (Figure 5: Artificial feeding and fluids). Hospital records showed little change, with the majority undocumented (64% to 62%) and only small proportions documented for (29%) or against (7–9%) artificial feeding. GP records were sparse but showed fewer “unknown” entries (85% to 65%, *p* = 0.008), mainly due to more documentation against artificial feeding (5% to 25%), while affirmative documentation remained low (10%).

**Figure 5.**
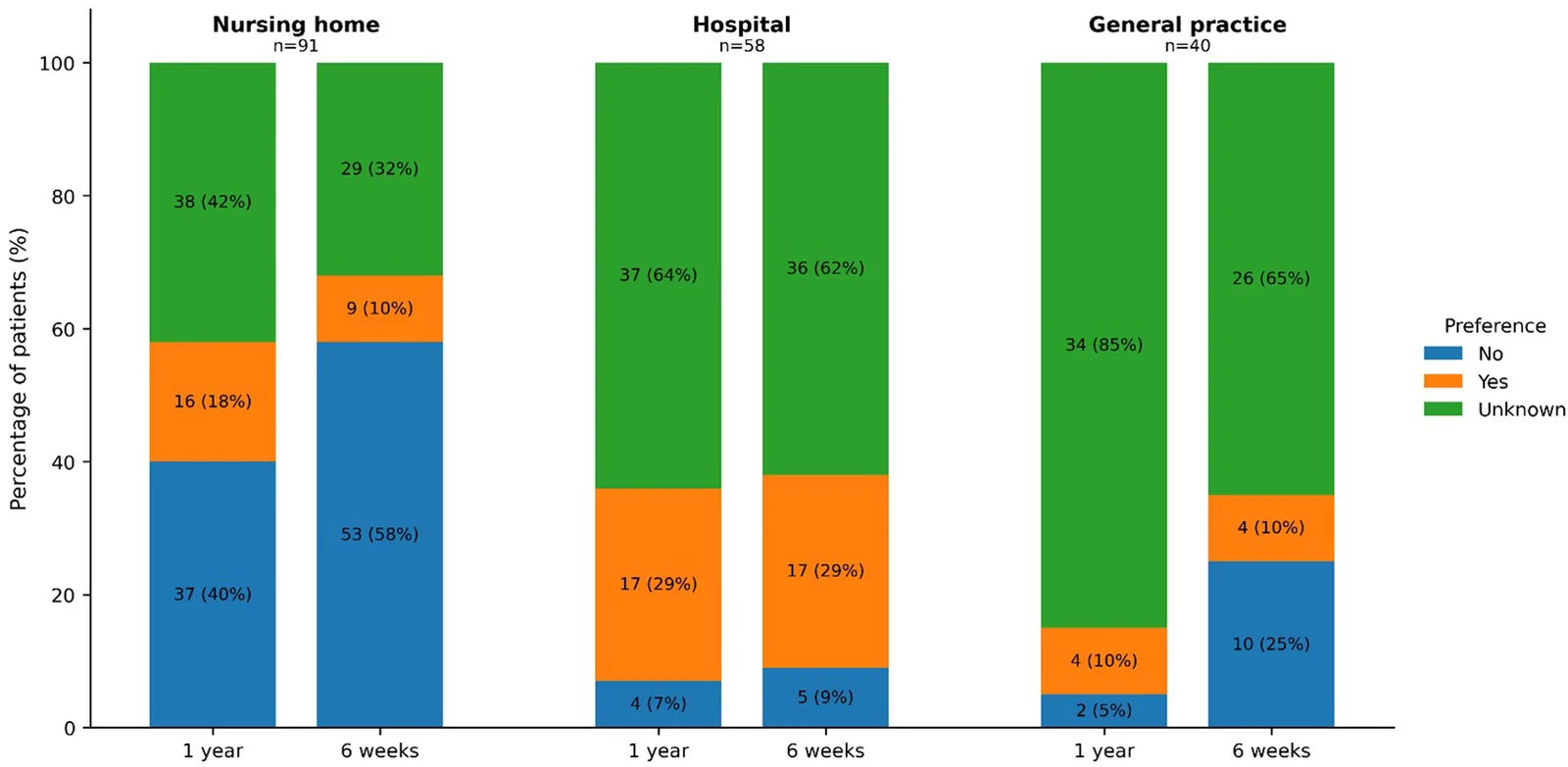
Artificial feeding and fluids.

In nursing home records, documented topics increased between one year and six weeks before death. This pattern was observed in discussions concerning disease-specific future scenarios (from 33.0% to 52.7%, *p* < 0.001), palliative sedation (from 3.3% to 17.6%, *p* < 0.001), and end-of-life issues (from 23.1% to 62.6%, *p* < 0.001) (Table 3). Although there were increases in discussions about preferred place of death (from 9.9% to 16.5%) and other treatment preferences (from 17.6% to 28.6%), these were not statistically significant.

**Table 3.** Topics of conversations documented in medical records.

	Nursing home n = 91	Hospital n = 58	General practice n = 40
Disease specific future scenarios - 1 year	30 (33.0)	19 (32.8)	6 (15.0)
- 6 weeks	48 (52.7)	38 (65.5)	10 (25.0)
P value difference between 1 year and 6 weeks	<0.001*	<0.001*	0.219
Preferred place of death - 1 year	9 (9.9)	3 (5.2)	7 (17.5)
- 6 weeks	15 (16.5)	9 (15.5)	14 (35.0)
P value difference between 1 year and 6 weeks	0.180	0.031	0.016*
Conversation about euthanasia - 1 year	16 (17.6)	3 (5.2)	14 (35.0)
- 6 weeks	19 (20.9)	3 (5.2)	16 (40.0)
P value difference between 1 year and 6 weeks	0.375	1.000	0.500
Conversation about palliative sedation - 1 year	3 (3.3)	1 (1.7)	8 (20.0)
- 6 weeks	16 (17.6)	9 (15.5)	13 (32.5)
P value difference between 1 year and 6 weeks	<0.001*	0.008*	0.125
Conversation about end-of-life - 1 year	21 (23.1)	12 (20.7)	6 (15.0)
- 6 weeks	57 (62.6)	29 (50.0)	8 (20.0)
P value difference between 1 year and 6 weeks	<0.001*	<0.001*	0.500
Other preferences for treatment - 1 year	16 (17.6)	1 (1.7)	11 (27.5)
- 6 weeks	26 (28.6)	6 (10.3)	12 (30.0)
P value difference between 1 year and 6 weeks	0.052	0.125	1.000

Exact p-values were used. *Significant after Bonferroni correction (critical p-value = 0.017).

In hospital records a similar trend was observed: documentation of disease-specific scenarios rose from 32.8% to 65.5% (*p* < 0.001), palliative sedation from 1.7% to 15.5% (*p* = 0.008), and end-of-life discussions from 20.7% to 50.0% (*p* < 0.001). Discussions on preferred place of death also increased (from 5.2% to 15.5%, *p* = 0.031).

General practice records showed smaller changes. The only significant increase was observed in discussions about preferred place of death (from 17.5% to 35.0%, *p* = 0.016). Other care discussions, including disease-specific scenarios, palliative sedation, and end-of-life topics, did not show significant changes. Euthanasia was documented relatively frequently at both time points (35.0% and 40.0%, respectively).

## Discussion

We found that across different healthcare settings, ACP decisions in persons with PD or AP predominantly concerned DNR resuscitation, ICU admission and hospital admission. However, the timing and extent of documentation varied substantially. In nursing homes, treatment preferences were recorded early and comprehensively. Almost all residents had “do not resuscitate” (DNR) and ICU limitation orders in place well before death, reflecting an established palliative orientation and a culture of anticipatory decision-making. In contrast, hospitals showed a sharp increase in documentation only during the final weeks of life. Decisions regarding DNR resuscitation, ICU care, and hospital admission were often made in response to acute deterioration. Several key topics remained underrepresented. Euthanasia was discussed relatively often in general practice but rarely in hospitals, while preferences regarding the place of death were documented for only a minority of patients overall.

In general practice records, ACP documentation remained sparse, even in the period close to death. Although discussions about future care may have taken place, they were poorly captured in the medical records—an important limitation also identified by Hommel et al. (2025), who conducted a medical record analysis of ACP conversations and diagnoses in Dutch general practice. Their study showed an incidence rate of first ACP documentation in PD of 19.1 per 1000 person-years, corresponding to approximately 6.1% of patients with PD having a recorded ACP conversation. Similarly, Bekker et al. (2022) showed that only 65% of Dutch GP records contained any ACP documentation, with markedly lower rates among patients with organ failure or multimorbidity compared to those with cancer.^18^ Both studies indicate that under-registration likely masks a higher prevalence of ACP discussions, yet together they highlight gaps in timely and structured recording of these conversations. This underlines the need for better integration of ACP in routine primary care documentation systems.

The limited scope of recorded preferences reflects previous research showing that ACP and advance directives (ADs) for people with PD often lack disease-specific relevance. Analyses of ADs from individuals with PD in Germany showed that while general medical decisions—such as DNR resuscitation or artificial feeding—were commonly included, PD-specific complications were largely absent.^19^ Key aspects such as the management of dopaminergic therapy, swallowing difficulties, cognitive decline, and neuropsychiatric symptoms were rarely addressed. Subsequent consensus work expanded on these observations by developing PD-specific recommendations to improve the clinical applicability of ADs.^20^ These recommendations emphasize the importance of explicitly incorporating PD specific issues.

Across all settings, the frequency of documented treatment preferences increased significantly toward the end-of-life, particularly for DNR status, ICU and hospital admission, antibiotic use, and artificial nutrition. This pattern indicates that decision-making is largely concentrated in the terminal phase,^21,22^ especially in nursing home settings where end-of-life care is more prominent. Although this temporal clustering may be explained by increasing medical complexity and proximity of death, it also raises important ethical and practical concerns. When discussions occur during episodes of acute deterioration, patients and families often face substantial time pressure and emotional distress, which may restrict opportunities for considered deliberation and shared decision-making.^4^ Moreover, cognitive impairment is common in advanced PD, further limiting patients’ capacity to participate in complex decisions regarding life-sustaining treatments. Consequently, it remains uncertain to what extent these late-stage decisions truly reflect patients’ own preferences, as opposed to proxy or clinician judgment.

These findings underscore the need for earlier and iterative ACP discussions, ideally initiated during stable disease phases before cognitive decline or acute illness constrains the patient's autonomy. A recent review advocates embedding ACP early within a neuropalliative framework—beginning soon after diagnosis and revisited at disease-related transition points such as functional decline, cognitive change, increasing dependence, or caregiver distress.^2^ The RADPAC-PD tool translates these principles into practice by identifying concrete clinical PD indicators for initiating ACP, including frequent falls, unplanned hospital admission, or a patient's expressed readiness.^23^ Integrating structured triggers into routine care may help shift ACP from a reactive, crisis-driven process to a proactive and person-centered approach, ensuring that decisions about treatment limitations and goals of care remain aligned with patients’ values throughout the disease course.

The documentation of palliative care goals differed between settings. When comparing diagnostic groups, patterns were largely similar for PD and AP. Physical goals were consistently prioritized, although documentation in AP cases tended to be slightly less frequent—particularly in hospitals. This suggests that differences in the scope of documented palliative care goals may be more closely associated with care setting than with the underlying diagnosis. Nursing home records most consistently reflected a multidimensional approach, encompassing physical, psychological, social, and existential domains. This holistic orientation aligns with the palliative care philosophy embedded in long-term care, where residents live until death and care focuses on comfort, dignity, and overall well-being rather than cure. In contrast, hospital documentation was almost exclusively limited to physical goals, with minimal attention to psychosocial or existential needs. The acute, treatment-centered environment of hospitals may prioritize medical interventions and symptom management, often at the expense of broader quality-of-life considerations. General practice records also showed limited attention to non-physical domains, despite the longstanding nature of GP–patient relationships. Strengthening the multidimensional scope of palliative care across all settings—addressing psychological, social, and existential dimensions alongside physical care—could promote a more consistent, person-centered palliative approach for people with PD and AP.

In conclusion, ACP documentation for people with PD and AP was inconsistent and often concentrated in the terminal phase, particularly for discussions that take place in healthcare settings other than nursing homes. Documentation focused mainly on treatment limitations such as ICU admission and DNR resuscitation, while broader psychosocial and disease-specific aspects were rarely addressed. Strengthening early ACP—supported by neurologist and PD nurse involvement and PD-specific guidance—may help ensure that end-of-life decisions more fully reflect patients’ values, medical realities, and evolving needs throughout the disease trajectory.

### Implications for practice

ACP for persons with people with PD or AP should start early and be revisited throughout the disease, allowing patients to express their preferences before cognitive decline limits decision-making. Tools such as RADPAC-PD can help identify appropriate moments for discussion.^23^ ACP should move beyond general treatment limitations to include PD-specific issues such as dopaminergic therapy, swallowing difficulties, and neuropsychiatric symptoms. Involving neurologists and PD nurse specialists alongside general practitioners can ensure that preferences are clinically feasible and aligned with disease progression. To achieve this, healthcare professionals need training to confidently address sensitive topics such as palliative sedation and euthanasia.^15^ Embedding ACP prompts in medical records and ensuring continuity across care settings may help transform ACP into a proactive, iterative process rather than a late, crisis-driven intervention, better aligning care with patient values throughout the disease trajectory.

### Limitations

First, the retrospective design limits interpretation of whether documented ACP reflects prospectively conducted advance care planning, in-the-moment decision-making during acute deterioration, or documentation entered after a crisis had already occurred. As a result, it is not possible to distinguish between anticipatory ACP and reactive, crisis-driven care planning, nor to infer causal relationships between the timing of ACP, the decisions made, and subsequent outcomes. In addition, the study period (2018–2023) overlapped with the COVID-19 pandemic, which may have influenced ACP practices and documentation. Previous studies suggest that the pandemic increased awareness of ACP and stimulated discussions about treatment preferences and goals of care.^24,25^ However, our sample size did not allow meaningful comparisons between the pre-pandemic and pandemic periods. Besides, some participating nursing homes and hospitals had been involved in the ParkinsonSupport project, which included education on palliative care in PD.^15^ Although ACP was addressed as part of this program, it was not the primary focus. Nevertheless, prior exposure may have increased awareness of ACP among healthcare professionals and influenced documentation practices, potentially leading to some overestimation of ACP documentation.

Second, patients were categorized according to their place of death and the corresponding source of the medical record. Consequently, we did not reconstruct complete care trajectories across healthcare settings, and ACP information transferred between settings or influenced by care transitions may not have been captured. Therefore, our findings reflect ACP documentation within the setting of death rather than longitudinal ACP trajectories.

Third, documentation quality varied across settings: while hospital and nursing home records included multidisciplinary input, general practice data were limited, likely underestimating ACP activity. Fourth, missing or incomplete entries may have led to underreporting, as ACP conversations are not always documented in medical records. Differences in record structures and terminology across settings may also have affected comparability. In addition, a small number of patients were classified as having *parkinsonism not otherwise specified/unclear* because the specific subtype could not be reliably determined from the medical records. Although these cases were presumed to represent progressive parkinsonian disorders, we cannot exclude the possibility that a small number had a non-neurodegenerative form of parkinsonism, such as drug-induced parkinsonism. Finally, this study focused on describing ACP documentation patterns rather than examining associations between patient characteristics and ACP documentation. Factors such as comorbidity, cultural background, social support, cognitive status, disease severity, and healthcare utilization may influence ACP engagement and documentation and should be explored in future research. However, the use of a structured data extraction form and regular consensus meetings between reviewers helped ensure consistency in data interpretation and minimize inter-rater variability, thereby strengthening the reliability of the findings despite these limitations.

## Data Availability

The data that support the findings of this study are available from the corresponding author upon reasonable request. The data are not publicly available.
